# Cost of the quanta SC+ hemodialysis system for self‐care in the United Kingdom

**DOI:** 10.1111/hdi.12994

**Published:** 2022-01-09

**Authors:** Thomas W. Ferguson, Gerard D. Harper, John E. Milad, Paul V. J. Komenda

**Affiliations:** ^1^ Department of Nephrology Seven Oaks Hospital Chronic Disease Innovation Centre Winnipeg Manitoba Canada; ^2^ Quanta Dialysis Technologies Limited Alcester UK

**Keywords:** cost effectiveness, dialysis, economic analysis, hemodialysis

## Abstract

**Introduction:**

New personal hemodialysis systems, such as the quanta SC+, are being developed; these systems are smaller and simpler to use while providing the clearances of conventional systems. Increasing the uptake of lower‐intensity assistance and full self‐care dialysis may provide economic benefits to the public health payer. In the United Kingdom, most hemodialysis patients currently receive facility‐based dialysis costing more than £36,350 per year including patient transport. As such, we aimed to describe the annual costs of using the SC+ hemodialysis system in the United Kingdom for 3×‐weekly and 3.5×‐weekly dialysis regimens, for self‐care hemodialysis provided both in‐center and at home.

**Methods:**

We applied a cost minimization approach. Costs for human resources, equipment, and consumables were sourced from the dialysis machine developer (Quanta Dialysis Technologies) based upon discussions with dialysis providers. Facility overhead expenses and transport costs were taken from a review of the literature.

**Findings:**

Annual costs associated with the use of the SC+ hemodialysis system were estimated to be £26,642 for hemodialysis provided 3× weekly as home self‐care; £30,235 for hemodialysis provided 3× weekly as self‐care in‐center; £29,866 for hemodialysis provided 3.5× weekly as home self‐care; and £36,185 for hemodialysis provided 3.5× weekly as self‐care in‐center.

**Discussion:**

We found that the SC+ hemodialysis system offers improved cost‐effectiveness for both 3×‐weekly and 3.5×‐weekly self‐care dialysis performed at home or as self‐care in‐center versus fully assisted dialysis provided 3× weekly with conventional machines in facilities.

## INTRODUCTION

The incidence and prevalence of kidney failure are rising, attributable to an aging population and the increased occurrence of comorbid conditions such as diabetes and hypertension.^1^ Most patients who experience kidney failure receive life‐sustaining dialysis, usually 3× weekly in a facility with a hemodialysis machine. Patients may also perform hemodialysis at home as self‐care or with a trained care partner. Recent trends, particularly in the United States, Canada, Australia, New Zealand, and the United Kingdom, indicate more frequent prescription of home hemodialysis,^1^, ^2^, ^3^, ^4^ with consistently demonstrated equivalent or improved outcomes in terms of cardiovascular events, mortality, and health‐related quality of life.^5^

Several barriers to the widespread uptake of home dialysis have been identified. These include medical contraindications (e.g., impaired manual dexterity, frailty, cognitive impairment), fear of self‐cannulation, inadequate space in the home, or a reluctance to medicalize the home setting.^6^, ^7^ The SC+ hemodialysis system is a novel hemodialysis machine developed by Quanta Dialysis Technologies (Alcester, United Kingdom) to address some of these barriers and make self‐care hemodialysis more accessible. The machine has been shown to be simple to operate with minimal training and has demonstrated a high level of user safety. It has a considerably more compact form factor than conventional dialysis machines and is easier to operate, store, clean, and maintain.^8^ The SC+ hemodialysis system has also been designed to be effective across a variety of dialysis prescriptions using conventional high‐flux dialyzers and dialysate flow rates. This allows patients to transition seamlessly between in‐center and at‐home settings under a variety of regimens from standard 3×‐weekly dialysis to more intensive daily or nocturnal regimens.^9^

Hemodialysis provided in a dialysis facility with full care from nursing staff is estimated to cost more than £24,000 (2009 British pounds—approximately £32,500 in 2019 British pounds) per patient, per year. In addition, the UK NHS (National Health Service) spends a further £50 million British pounds annually on patient transport for dialysis treatment (approximately £68 million in 2019 British pounds).^10^ Home hemodialysis with conventional machines has consistently been shown to offer improved cost‐effectiveness compared with facility‐based treatment, reducing overall costs by one‐quarter to one‐third.^11^, ^12^, ^13^ Dialysis provided at home also allows patients greater flexibility in their treatment schedule, improved quality of life and satisfaction, and the ability to perform more frequent dialysis prescriptions at substantially reduced costs compared with facility‐based hemodialysis.^14^ These more frequent prescriptions may help mitigate the increased hospitalization and emergency department use associated with the long interdialytic gap experienced over the weekend under a 3×‐weekly hemodialysis regime.^15^

As the quanta SC+ hemodialysis system has demonstrated improved ease of use in human factors testing,^8^ we aimed to describe the costs associated with both using the machine for self‐care in‐center and in the home, for both 3×‐weekly and 3.5×‐weekly (every other day) hemodialysis regimens.

## MATERIALS AND METHODS

A cost minimization model was constructed following generally accepted guidelines for economic evaluation.^16^ All costs are presented in 2019 British pounds (GBP), and historical estimates taken from the literature were inflated using estimates of inflation from the Bank of England.^17^ As no personal health data were used in deriving the cost estimates, approval from an institutional ethics board was not sought. The model was constructed using Microsoft Excel for Office 365.

The primary outcome of interest was the annual direct cost for dialysis provided with the SC+ hemodialysis system at two levels of treatment intensity (3× and 3.5× weekly) in its main intended use cases: self‐care in‐center and self‐care at home. The model presents the annual dialysis cost; therefore, the application of discount rates was not required.

The model was constructed from the perspective of the UK public health payer and included component costs associated with the management and delivery of dialysis that are reimbursed by the UK NHS, either as part of the renal program reimbursement bundle, or as part of the broader health system. The first component was human resource costs (nursing, renal technicians, dieticians, pharmacists, social workers, and counselors), which were calculated using anticipated staffing ratio estimates from the device developer (Quanta Dialysis Technologies) and assuming that nursing staff would be compensated at the second highest band of the NHS pay scale. Human resource expenses were assessed on a per‐treatment basis and greater detail on these costs is provided in Supplementary Item S1 and were sourced based on discussions with dialysis providers. This assumed a 60:1 ratio of nurses to patients for the self‐care at home modality, and a ratio of 7:1 for self‐care in‐center. Benefits and an allowance for vacation time were assessed at 20% of the total direct human resource expenses, and sick time and relief were assessed at an additional 20%.^13^

The costs of dialysis‐related consumables and equipment were also sourced from the device developer and were based on the NHS Supply Chain reference prices. This cost element included all expenses related to both proprietary and nonproprietary dialysis consumables, the provision of the dialysis machine, the reverse osmosis water filtration machine in the home setting, delivery of consumables to the home or to the center, and machine maintenance and service. The total bundle price is £124.00 per treatment in 2019–2020 for a patient receiving dialysis as self‐care at home. For patients receiving dialysis as self‐care in‐center, only a portion of the dialysis machine and machine service costs were allocated to that patient, assuming four patients would be able to use a station for dialysis provided 3× weekly as self‐care in‐center, and that 3 patients would be able to use a station for dialysis provided 3.5× weekly as self‐care in‐center. Estimates of dialysis‐related clinic overhead (including hospital utilities) were taken from a previously published multicenter study of the cost of dialysis in the United Kingdom, totaling £7563.26 for a patient receiving dialysis 3× weekly in clinic, and £8823.80 for a patient receiving dialysis 3.5× weekly.^18^ For patients who receive therapy at home, we also assumed that there would be some attributed clinic space with associated overhead, totaling £422.77 annually.^18^

Patients receiving dialysis in the home also require periodic visits to a facility‐based dialysis unit for respite, ongoing training, and other medical care (e.g., social work, dietician appointments). The model therefore included an additional cost for home dialysis patients representing in‐center runs requiring use of a conventional dialysis unit. This was assumed to average 11 runs per home dialysis patient per year based on published experience with large home hemodialysis programs.^11^ Estimates from a previously published multicenter study of dialysis costs in the United Kingdom were used to derive the cost associated with an in‐center dialysis run. When these estimates were inflated to 2019 GBP, the total annual cost of in‐center conventional hemodialysis was £32,500, providing a per‐run estimated cost of £208.^18^ The clinic overhead related to home hemodialysis was assumed to be included within these in‐center run expenses.

The UK NHS funds approximately 61% of patient journeys to and from clinic‐based hemodialysis. Accordingly, transport expenses were included as a component of the cost model and totaled £3773.34 annually for a conventional 3×‐weekly dialysis prescription. This figure was derived from a 2010 NHS audit and inflated to 2019 GBP.^10^ An annual transportation expense was also added for home dialysis patients for the assumed 11 in‐center runs.

A detailed overview of model assumptions is provided in Table 1.

**TABLE 1:** Overview of model assumptions and sources

Model input	Definition	Amount	Source
Human resources	Cost per treatment	£20 (home self‐care 3× weekly) £17 (home self‐care 3.5× weekly)£21 (self‐care in‐center 3× weekly) £18 (self‐care in‐center 3.5× weekly)	Supplemental Item S1, Communication with Quanta Dialysis Technologies and dialysis providers
Benefits	Percentage of HR expense	20%	Assumption
Vacation, sick time, and relief hours	Percentage of HR expense	20%	Assumption
Dialysis‐related consumables and equipment	Cost per treatment	£124.00	Communication with Quanta Dialysis Technologies
Hospital overhead (self‐care in‐center only)	Cost per year	£7563.26 (in center, 3× weekly) £8823.80 (in‐center, 3.5× weekly) £422.22 (home self‐care)	Baboolal et al.^18^
In‐center runs	Cost per treatment(assumes 11 runs annually)	£208.00	Komenda et al.,^11^ Baboolal et al.^18^
Dialysis‐related transportation	Cost per year	£266.00 (home self‐care) £3773.34 (self‐care in‐center)	Kerr et al.^10^

Many of the cost components included in the model were not presented with measures of variation, but would be expected to experience variance based on the location or center where the service is delivered, and as such we varied human resources, clinic overhead, and transportation expenses with an increase of 25% from baseline to assess their potential impact on overall cost, with the assumption that equipment and consumables expenses would remain relatively constant between centers.^11^

Lastly, as there is heterogeneity between health systems and centers with respect to reimbursement for utilities related to dialysis provided in a patient's home, a scenario analysis was performed to account for the incremental electrical and water‐related utility costs associated with home therapy. Utility costs assumed that the machine would use 10.36 kw/h and approximately 288 liters of water per treatment. Using these assumptions, electricity costs would total approximately £2 per treatment,^19^, ^20^ and the water cost would be approximately £2.28 per cubic meter (1 m^3^ = 1000 L) for supply and sewerage, with a standing charge of £0.47 per treatment.^21^

## RESULTS

Annual costs associated with the use of the SC+ hemodialysis system were estimated to be £26,642 for hemodialysis provided 3× weekly as home self‐care; £30,235 for hemodialysis provided 3× weekly as self‐care in‐center; £29,866 for hemodialysis provided 3.5× weekly as home self‐care; and £36,185 for hemodialysis provided 3.5× weekly as self‐care in‐center. A summary of annual costs by dialysis modality and frequency of treatment is provided in Table 2.

**TABLE 2:** Annual per‐patient cost for 3× and 3.5× weekly prescriptions with the SC+ hemodialysis system for hemodialysis provided as self‐care at home and self‐care in‐center

	3× weekly self‐care home	3× weekly self‐care in‐center	3.5× weekly self‐care home	3.5× weekly self‐care in‐center
Human resources				
Nursing	£1969	£2134	£1969	£2134
Renal technician	£415	£415	£415	£415
Social work	£200	£200	£200	£200
Dietician	£170	£170	£170	£170
Counselor	£292	£292	£292	£292
Pharmacy	£39	£39	£39	£39
Total direct human resources	£3085	£3250	£3085	£3250
Benefits, vacation, relief, and sick time	£1234	£1300	£1234	£1300
Dialysis consumables and equipment	£19,344	£14,349	£22,568	£18,410
Clinic overhead	£423	£7563	£423	£8824
In‐center runs	£2292	N/A	£2292	N/A
Transportation	£266	£3773	£266	£4402
*Total*	*£26,642*	*£30,235*	*£29,866*	*£36,185*

**FIGURE 1:**
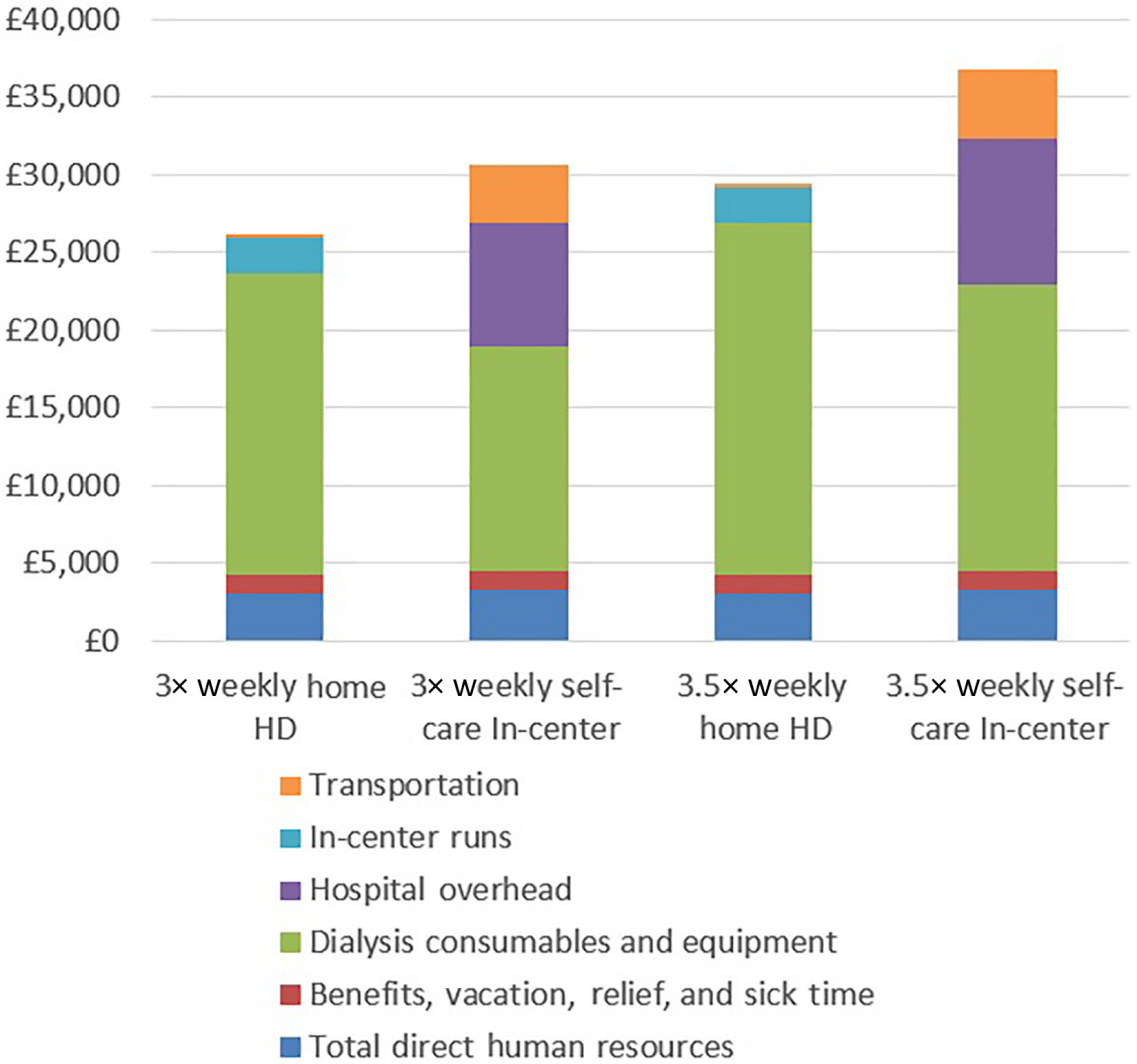
Overview of component costs by location and frequency of dialysis treatment [Color figure can be viewed at wileyonlinelibrary.com]

An overview of the relative magnitude of component costs is provided in Figure 1. The primary cost driver was dialysis consumables and equipment, representing between 47% and 51% of total expenses for patients receiving dialysis as self‐care in‐center, and between 73% and 76% of expenses for patients receiving dialysis at home. For patients receiving in‐center treatment, influential cost drivers included clinic overhead, anticipated to be approximately £7563 for patients receiving treatment 3× weekly and £8824 for patients receiving therapy 3.5× weekly, representing about 25% of total expenses. Transportation expenses represented approximately 12% of the total treatment cost for patients in‐center, totaling £3773 for patients receiving 3×‐weekly dialysis and £4402 for patients receiving 3.5×‐weekly dialysis. Human resources cost as a proportion of total treatment cost was similar across modalities at between 12% and 16%, including benefits, vacation, sick time, and relief.

In our univariate sensitivity analysis increasing human resource expense by 25%, we found that total costs for therapy delivered 3× weekly reached £27,721 for home self‐care, and £31,372 for self‐care in‐center, and for the 3.5×‐weekly regimen costs reached £30,946 for home self‐care and £37,322 for self‐care in‐center. For clinic overhead expenses, a 25% increase provided an annual dialysis cost of £32,125 for the 3×‐weekly regimen and £38,391 for the 3.5×‐weekly regimen, and had a negligible effect on expenses for self‐care at home modalities with a total of £26,745 for 3× weekly self‐care at home, and £29,972 for 3.5× weekly. Transport costs were a relatively minor expense for patients receiving therapy at home, but for patients performing dialysis as self‐care in‐center, an increase of 25% in transport costs raised total expenses to £31,178 for the 3×‐weekly regimen and £37,286 for 3.5×‐weekly regimen.

In our scenario analyses examining utility costs we found that annual electrical costs were estimated to be £312 for patients receiving 3×‐weekly dialysis, increasing to £364 for patients receiving 3.5×‐weekly therapy. Similarly, annual water expense totaled £176 for patients receiving 3×‐weekly dialysis, increasing to £205 for patients receiving 3.5×‐weekly therapy. Total utility expense was not a major cost driver, representing less than 2% of overall annual expense.

## DISCUSSION

In this cost minimization analysis, we provide an overview of the overall costs associated with self‐care hemodialysis undertaken at home and as self‐care in‐center using the SC+ hemodialysis system in the United Kingdom. In these settings, annual dialysis‐related treatment cost ranged between approximately £26,000 and £36,000 for 3×‐ and 3.5×‐weekly regimens. Our analysis also confirms other studies from different countries demonstrating that dialysis provided in a patient's home, either with hemodialysis machines or as peritoneal dialysis, is a cost‐effective alternative to facility‐based dialysis.^11^, ^12^, ^13^, ^22^ Furthermore, this study provides an evaluation of a new dialysis machine, the SC+ hemodialysis system, and describes the costs associated with self‐care in‐center dialysis.

The most recent detailed analysis of the costs of chronic kidney disease to the UK NHS, performed prior to the introduction of the current structure of dialysis reimbursement, found in‐clinic hemodialysis to cost approximately £32,500 for direct treatment, and £3773 for transportation (total, £36,273) after accounting for inflation.^18^ Our findings suggest that, from a payer perspective, the SC+ hemodialysis system is a cost‐effective alternative to conventional dialysis machines for self‐care both at home and in a hemodialysis facility. Moreover, our study described the costs of human resources as representing roughly 7% of the total maintenance treatment cost, versus over 50% in facility‐based modalities in recent studies.^13^, ^18^

Home‐based dialysis therapies often require greater patient autonomy than those provided under the full supervision of nursing staff, and there are concerns that their increased use may pose a risk to patient safety. However, systematic reviews of studies of the effectiveness of home‐based therapies have consistently found either a decrease in mortality, or similar mortality, when compared with conventional dialysis provided in‐center.^5^ Additionally, randomized trials evaluating more frequent home hemodialysis have shown benefits in terms of clinically important cardiovascular outcomes (left ventricular mass index regression) and improved quality of life, although these trials were small and of short duration in carefully selected patients.^23^

Decisions regarding policy on home dialysis therapies must also account for findings from observational studies. For example, a trial in the Netherlands that attempted to randomize eligible patients to either home therapy or in‐center therapy after providing education and fully informed consent found that more than 95% of patients had a specific preference for either in‐center hemodialysis or home peritoneal dialysis and chose to not be randomized.^24^ Nonetheless, rigorously performed observational studies across multiple populations have been consistent in demonstrating a low risk of harm, and plausible benefits.^25^, ^26^

Dialysis provided more frequently than 3× weekly in clinic with full nursing support has been shown to offer marginal cost‐effectiveness versus conventional 3×‐weekly dialysis, with cost‐utility ratios between $75,000/quality‐adjusted life year (QALY) and $125,000/QALY (approximately £60,000/QALY–£100,000/QALY). Much of the incremental cost associated with more frequent dialysis is attributable to the additional human resources and medical supplies required.^27^ With dialysis provided in a patient's home, however, the costs associated with additional skilled human resources can be avoided and previous economic evaluations have consistently shown that nocturnal home hemodialysis or daily home hemodialysis offer both reduced costs and improved or similar quality of life compared with full‐service in‐center dialysis.^14^, ^28^

There are further benefits that may be realized by switching to more frequent home and self‐care in‐center hemodialysis prescriptions. A recent study of over 240,000 patients with kidney failure in the United States Renal Data System described an “interdialytic‐gap effect” associated with the 2‐day break at weekends experienced by patients receiving 3×‐weekly dialysis prescriptions. The study found a significant increase in both emergency department visits and hospitalizations following the weekend regardless of whether patients received a Monday–Wednesday–Friday or a Tuesday–Thursday–Saturday schedule.^15^ Similar findings have been shown in a cohort of patients from the United Kingdom Renal Registry, with a 70% increase in hospital admission rates following the 2‐day gap, and a 20% increase in mortality.^29^ Additionally, a registry study of dialysis patients from a Canadian cohort demonstrated a 34% increase in emergency department visits on Monday and a 20% increase on Tuesday versus other days of the week among patients on a Monday–Wednesday–Friday schedule.^30^ In particular, it has been suggested that there may be a benefit in reducing cardiovascular‐related hospital admissions when comparing patients receiving more frequent home hemodialysis versus those on 3×‐weekly schedules.^31^ The 3.5×‐weekly (every other day) dialysis schedule modeled in this present analysis represents a scenario in which this 2‐day gap is eliminated.

Implications regarding patient uptake of home hemodialysis, including ease of training, patient retention, and relief during periods of hospitalization, also warrant further consideration with respect specifically to the SC+ hemodialysis system. Previous research has shown that home hemodialysis patients can be trained quickly to use simpler, compact dialysis machines^13^ and this can have a substantial effect on the sunk costs associated with initiating a new home hemodialysis patient. Ease of use also increases the population of patients capable of self‐care and allows for assisted home dialysis with less labor‐intensive delivery. Moreover, with respect to relief, the SC+ hemodialysis system has been designed to allow seamless transition between facility‐based and at‐home environments. In addition, our analysis shows that utility costs (electricity and water) in a patient's home are minor expenses within the total cost of providing dialysis. However, they may still be a barrier to the acceptance of home therapy by patients with lower socioeconomic stability, and we recommend that renal programs consider introducing reimbursement systems to alleviate this barrier if they are not currently in place.^32^ It is also important to note that the cost per patient with respect to clinic‐based utility expenses is often difficult to determine as these expenses are usually only reported in total overhead expenses.

This analysis has several strengths. First, our model presents a granular breakdown of costs related to the SC+ hemodialysis system, and as such provides a framework by which policy makers can judge the impacts of changing dialysis modality and equipment on specific budgetary items. Second, we incorporate the cost of transportation to and from dialysis in our model. Dialysis‐related transportation costs the UK NHS over £50 million annually, and the United States has reported that increases in transport expenses to Medicare are outpacing other expense items, with annual per‐patient costs estimated between $7000 and $10,000 (approximately £5500–£8000).^10^, ^33^

This study also had several limitations. As we take the perspective of the public health payer, there are some potential costs from a societal perspective that may not be reflected in our estimates. These include changes to caregiver burden, modifications to the home to enable home dialysis that are not covered by healthcare systems, and changes to productivity because of more frequent dialysis prescriptions. Also, as the SC+ hemodialysis system is at the beginning of its commercial introduction, large‐scale primary data are not yet available to derive more estimates of uncertainty around costing estimates. As the system begins to achieve scale in delivery of its service offering and manufacturing capabilities, its unit costing would be expected to decrease. As such, this evaluation presents an initial benchmark that policy makers can use to inform early adoption of the new technology. Where costing inputs may vary between different dialysis programs, we have tried to address this with our sensitivity analysis exercise, in particular human resource expenses where different programs may employ staff with differing levels of experience. Lastly, as this analysis only provides an overview of maintenance dialysis expenses, the initial training expenses associated with preparing a patient for self‐care dialysis have not been evaluated; however, costs associated with patient training on SC+ are expected to be substantially lower than those for traditional machines due to the ease of use of the SC+ platform which was developed with the intended use by patients.^8^

In conclusion, this cost minimization analysis demonstrates that the SC+ hemodialysis system offers improved cost‐effectiveness for both 3×‐weekly and 3.5×‐weekly (every other day) self‐care dialysis performed at home or as self‐care in‐center versus fully assisted dialysis provided 3× weekly with conventional machines in facilities. Further cost‐effectiveness analyses incorporating more frequent dialysis prescriptions (e.g., short daily) and their potential related benefits to reducing hospital and emergency department visits are needed to understand fully the economic benefits of increasing home dialysis prescriptions.

## CONFLICT OF INTEREST

Gerard Harper and John E. Milad are employees of Quanta Dialysis Technologies. Dr. Paul Komenda and Thomas Ferguson are consultants to Quanta Dialysis Technologies. This study was funded by Quanta Dialysis Technologies.

## Supporting information


**Data S1**: Per treatment human resources expenses.
