# Supplementary material for: Cost of the quanta SC+ hemodialysis system for self‐care in the United Kingdom
Source: Hemodial Int. 2022 Jan 9;26(3):287–94. doi: 10.1111/hdi.12994 (PMC9544577; doi:10.1111/hdi.12994)
Supplement: Supplementary file 1 — Data S1: Per treatment human resources expenses. [file HDI-26-287-s001.pdf]

Supplement

Item S1. Per treatment human resources expenses

	**3x weekly self-care home**	**3x weekly self-care in-center**	**3.5x weekly self-care home**	**3.5x weekly self-care in-center**
Nursing	£12.62	£13.68	£10.82	£11.73
Renal technician	£2.66	£2.66	£2.28	£2.28
Social work	£1.28	£1.28	£1.10	£1.10
Dietician	£1.09	£1.09	£0.93	£0.93
Counselor	£1.87	£1.87	£1.60	£1.60
Pharmacy	£0.25	£0.25	£0.21	£0.21
Total number of treatments per year	156	156	182	182
**Total cost per treatment**	**£19.77**	**£20.83**	**£16.94**	**£17.85**

3.5x weekly dialysis assumes that pharmacy, counselor, dietician, and social work requirements are the same regardless of treatment frequency. Nursing and renal technician are expensed per treatment
